# Optical Switching of Robust Ferroelectric Polarization on Epitaxial Hf_0.5_Zr_0.5_O_2_ Integrated with BaTiO_3_

**DOI:** 10.1007/s40820-026-02090-2

**Published:** 2026-02-06

**Authors:** Wenjing Dong, Huan Tan, Jingye Zou, Alberto Quintana, Tingfeng Song, César Magén, Claudio Cazorla, Florencio Sánchez, Ignasi Fina

**Affiliations:** 1 Institut de Ciència de Materials de Barcelona (ICMAB-CSIC), Campus UAB, 08193 Bellaterra, Barcelona, Spain; 2 Departament de Física, Universitat Autònoma de Barcelona, 08193 Cerdanyola del Vallès, Spain; 3 Instituto de Nanociencia y Materiales de Aragón (INMA), CSIC-Universidad de Zaragoza, 50009 Saragossa, Spain; 4 Departamento de Física de la Materia Condensada, Universidad de Zaragoza, 50018 Saragossa, Spain; 5 Group of Characterization of Materials, Departament de Física, Universitat Politècnica de Catalunya, Campus Diagonal Besòs, Av. Eduard Maristany 10–14, 08019 Barcelona, Spain; 6 Research Center in Multiscale Science and Engineering, Universitat Politècnica de Catalunya, Campus Diagonal-Besòs, Av. Eduard Maristany 10–14, 08019 Barcelona, Spain; 7 Institució Catalana de Recerca I Estudis Avançats (ICREA), Passeig Lluís Companys 23, 08010 Barcelona, Spain

**Keywords:** HfO_2_, Hafnium oxide, Multilayers, Hf_0.5_Zr_0.5_O_2_, Ferroelectric, Optoelectric

## Abstract

Optical switching of ferroelectric polarization is of interest for wireless and energy-efficient control of logic states. So far, this phenomenon has been widely demonstrated only in ferroelectric perovskites, while studies on other emerging ferroelectrics remain limited. In this regard, the paradigmatic example of a technologically relevant ferroelectric material is HfO_2_. However, HfO_2_ has a very wide bandgap, limiting light absorption. So far, the proposed strategies to enhance light absorption in HfO_2_-based systems are detrimental to ferroelectric properties, i.e., bandgap lowering or on-purpose defect introduction, which reduce switchable polarization and increase the presence of leakage currents. Here, we show that good ferroelectric properties, i.e., sizeable polarization (up to 15 μC cm^−2^), low leakage current (under 10^–6^ A cm^−2^), high endurance (up to 10^8^ cycles) and fast switching (< 50 ns), can be achieved in epitaxial Hf_0.5_Zr_0.5_O_2_ films through an alternative strategy, BaTiO_3_ capping. While ferroelectric properties are remarkable, we demonstrate that the presence of BaTiO_3_ allows light absorption and the concomitant electric field generation, as supported by density functional theory calculations, which enables optical switching of polarization in Hf_0.5_Zr_0.5_O_2_ under 405 nm illumination. It is observed that optical switching is more efficient in films with thicker BaTiO_3_ capping layer. The high polarizability of BaTiO_3_ contributes to minimizing degradation in the ferroelectric response of the system. The results presented here indicate that appropriate designs can be followed to obtain optical switching of polarization in ferroelectric HfO_2_ while preserving main functional properties.

**Supplementary Information:**

The online version contains supplementary material available at 10.1007/s40820-026-02090-2.

## Highlights

- Integration of perovskite BaTiO_3_ with epitaxial fluorite Hf_0.5_Zr_0.5_O_2_ is demonstrated.
- Polarization up to 15 ﻿μC cm^−2^, leakage current densities below 10^–6^ A cm^−2^, endurance up to 10^8^ cycles, and switching times shorter than 50 ns are achieved.
- Remote optical switching of the polarization is demonstrated, and it is shown to be controlled by the thickness of the BaTiO_3_ capping layer.

**Supplementary Information:**

The online version contains supplementary material available at 10.1007/s40820-026-02090-2.

## Graphical abstract

**Figure:**
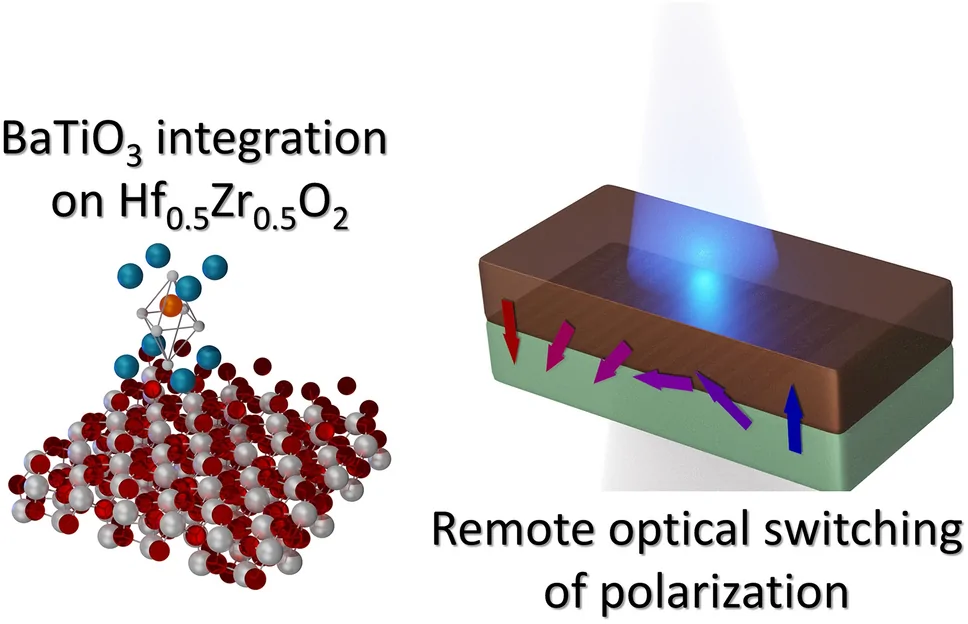

## Introduction

Ferroelectric memories have been on the market for more than 30 years. These are competitive in terms of power consumption, speed, retention, and endurance. However, these are based on Pb(Zr,Ti)O_3_ (PZT) [1], which has the drawbacks of toxicity and low memory density. The reason for the low memory density capability is that PZT does not allow 3D integration processes and the node size in memory devices based on it cannot be scaled down below ~ 130 nm [2, 3]. Among all the ferroelectric materials, the scientific community has recently focused its attention on ferroelectric hafnium oxide [4]. The reason is that it is the most robust ferroelectric material fully compatible with complementary metal oxide semiconductor (CMOS) technology, and thus improved memory density can be achieved. Remarkably, ferroelectric memory devices at commercialization stage have been already reported [5, 6]. So far, research on hafnium oxide has primarily concentrated on polycrystalline films [2, 3].

Memory devices based on ferroelectric materials aim to involve only electronic processes [7], which are intrinsically energy-efficient [8]. The combination of ferroelectric materials with magnetic materials [9] or the use of the inherent large photovoltaic response of ferroelectrics [10] has been reported to result in further reduction of energy consumption for both the writing and reading processes. Exclusive phenomena related to the presence of ferroelectric polarization and its interaction with light are large photostrictive coefficients [11] and large open-circuit voltages [12]. Photostriction in ferroelectric/magnetic heterostructures has been used to modify magnetic properties with light [13–15]. Additionally, optoelectronic devices leveraging the presence of internal electric fields generated by ferroelectric polarization [16, 17] and memory cells based on light-controlled-polarization-dependent photoconductance [10, 18] have been developed. Recently, it has been shown that the population of polar domains with different orientations and associated domain walls in ferroelectric multidomain BaTiO_3_ (BTO) single crystals, can be modulated by suitable coherent illumination [19, 20] or ultrafast light pulses [21], giving an additional path for investigation. The use of subsidiary devices [22, 23] has been reported to allow reversible optical switching of polarization. Light absorbed at semiconducting electrodes (e.g., MoS_2_) can also trigger polarization switching as demonstrated in three-terminal ferroelectric field-effect transistor [24, 25]. In two-terminal ultrathin BTO junctions, light has been demonstrated to modify the tunneling [26, 27] or Schottky-limited [28] current as a consequence of polarization switching. Therefore, optical switching of polarization is a feasible and extremely interesting strategy from an applications perspective. Unfortunately, none of the ferroelectric materials in which optical switching has been investigated in the past is CMOS-compatible. This calls for studies on optical switching in HfO_2_-based devices.

**Fig. 1:**
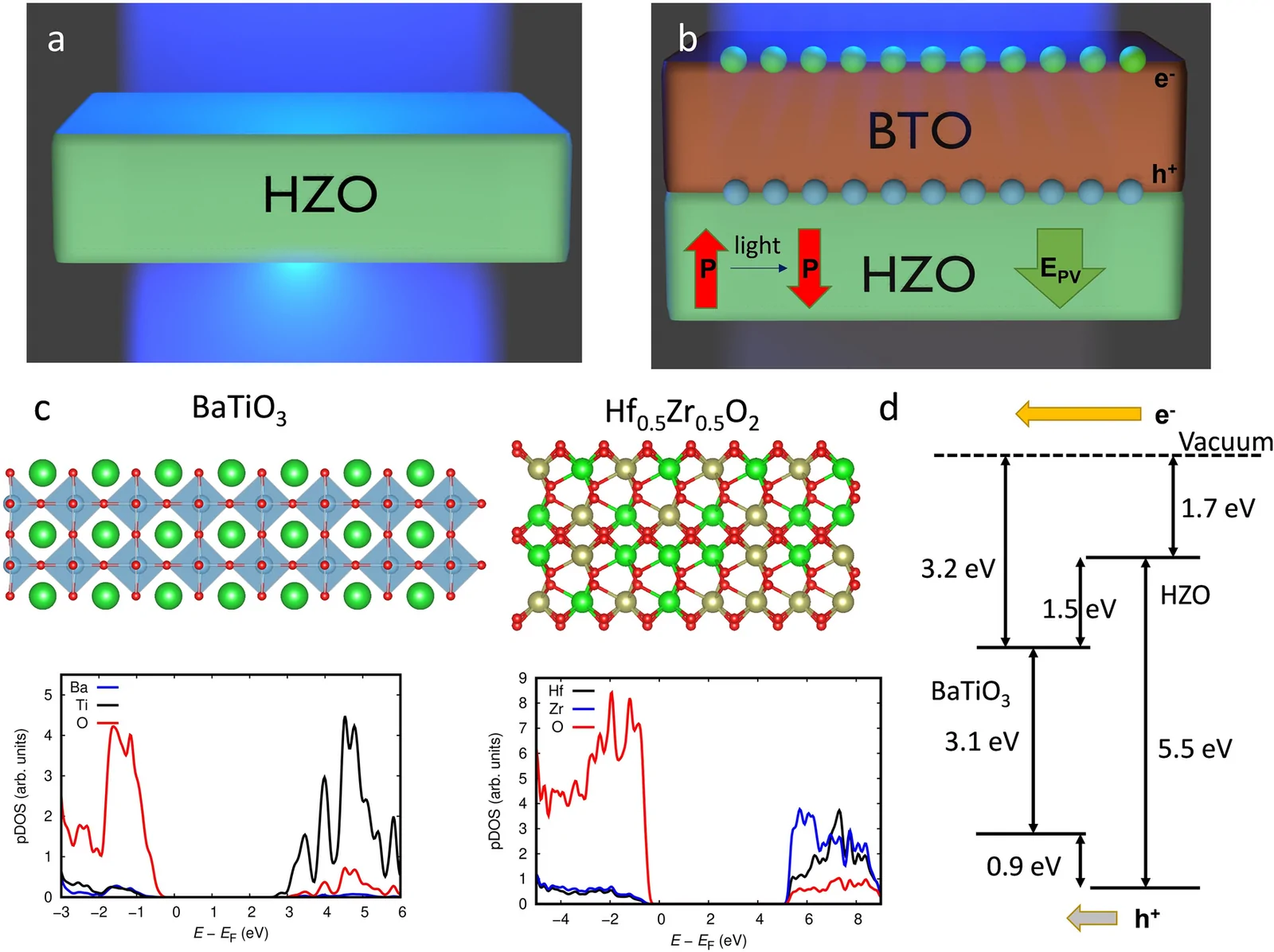
Schematic representation of **a** the transparency of the HZO layer under illumination with 3.06 eV light and **b** the partial absorption of light, generation of E_PV_ and switching of ferroelectric polarization in presence of the top BTO layer. **c** Sketch of the structures for BTO and HZO used in DFT calculations and obtained density of states. **d** Computed band diagram for the BTO/HZO junction from first-principles DFT calculations

Regarding optical control of ferroelectric polarization in hafnium oxide, it must be noted that its bandgap is wide [≈ 6.0 eV for Hf_0.5_Zr_0.5_O_2_ (HZO)] [29, 30], making it transparent to visible light (Fig. 1a) and therefore very few reports can be found on the effect of light in this material. Electrode, interfaces and/or defects [31–34] have been reported to allow light absorption. Similar is the case for ZrO_2_ [35]. Regardless of the strategy employed to enable photoabsorption, optical polarization switching in hafnia has been reported to be possible by light-induced internal electric fields [32]. However, in the reported devices polarization is near 2 μC cm^−2^, well below the polarization values required for applications (see Table 1). This is a consequence of the defective nature of the studied systems, which show visible light absorption at the expense of poorer ferroelectric properties. In addition, no reports on device endurance under illumination are available, which is a crucial technological parameter.

**Table 1:** Summary of remanent polarization (P_r_) values in different systems based on HfO_2_ or ZrO_2_ where optoelectric response has been characterized. It is also indicated if the optical switching of polarization has been observed and if endurance tests have been performed

System	P_r_ (μC cm^−2^)	Reliability test	Wavelength (nm)	Optical switching time	Refs.
Si//HfO_2_/AgNWs	2.5–4.96	no	940	NE	[31]
Nb:SrTiO_3_//HZO/Pt	1	no	405–648	1 s	[32]
Si//HfO_2_/Al:HfO_2_/Al_2_O_3_/Graphene/Au	1.92	no	532	Apparent*	[33]
Si//Si:HfO_2_/TiN	not given	no	AM1.5	NE	[34]
ITO//ZrO_2_/SiOx/Si/Al	1.2	no	940	NE	[35]
SrTiO_3_//LSMO/HZO/BTO/Pt	15	yes	405	∼ 1 s	Present work

*Apparent accounts for the observation of memory effects without direct demonstration of the optical switching. NE: not evaluated. AM1.5 denotes the standard terrestrial solar spectrum corresponding to an air mass of 1.5

Therefore, it is necessary to study the combination of absorbing materials with ferroelectric hafnia and to mitigate the detrimental impact on technological parameters. With this aim, we have combined epitaxial ferroelectric Hf_0.5_Zr_0.5_O_2_ (HZO) with a light absorbing material. In 2018, it was reported the epitaxial growth of ferroelectric hafnia on perovskite SrTiO_3_(001) (STO) substrates using pulsed laser deposition (PLD) [36, 37], with good retention and endurance and without the presence of prominent wake-up effects [38]. Epitaxial films, although not CMOS compatible, are of the highest interest for the understanding of the intrinsic properties of the material [39]. Despite the large lattice mismatch between HfO_2_ and La_0.67_Sr_0.33_MnO_3_ (LSMO), ferroelectric HfO_2_ grows with excellent properties on LSMO by the unconventional domain matching epitaxy mechanism [40]; however, this is not possible with other perovskite such as LaNiO_3_ or SrRuO_3_ [41]. Interest in perovskite materials within the scientific community has intensified during the last years [42–44]. Here, we demonstrate that BTO can be integrated on top of epitaxial HZO while preserving good crystalline quality for both layers and the system ferroelectric response, concomitantly with visible-light absorption enhancement. BTO has been chosen due to its lower coercive field (E_c_) compared to ferroelectric hafnium oxide. This makes BTO highly polarizable and therefore the presence of large depolarization fields is mitigated. At the same time the presence of photovoltaic effects in BTO using light in or near to the visible range is well-known [45–47]. In a HZO/BTO bilayer structure (Fig. 1b), light absorption at BTO and the concomitant presence of photovoltaic effect guarantees the generation of charges. These charges accumulate generating the presence of an additional electric field (E_PV_, directed downwards in the figure for convenience). The direction of this E_PV_ will dictate the final polarization direction from up to down in the Fig. 1b. Indeed, we performed first-principles calculations based on density functional theory (DFT) to obtain the band gaps of the BTO and HZO slabs depicted in Fig. 1c. To estimate band alignments, we performed DFT calculations in combination with the range-separated hybrid functional HSE06 [48], a computational approach described in previous works [49, 50], to obtain electronic configuration of BTO and HZO layers. In Fig. 1c, the density of states of both materials is also included. For BTO, the valence band (VB) is clearly dominated by O*-p*–type orbitals, which hybridize with Ti-*d* and Ba-*p*,*d* orbitals. On the other hand, the conduction band (CB) is dominated by Ti-*d*–type orbitals, which mainly hybridize with O-*p* orbitals. For HZO, the VB is also clearly dominated by O-*p*–type orbitals that hybridize with Hf-d orbitals. In addition, Zr-*d* orbitals participate together with Hf-d orbitals in the VB and dominate in the CB. In Fig. 1d, the resulting band alignment is depicted. It can be observed the presence of a straddling gap at the HZO/BTO interface. Under illumination, electron–hole pairs are generated in the heterostructure. Under open-circuit conditions, photogenerated carriers cannot flow through the external circuit; as a result, electrons are driven toward and accumulate at the BTO top surface. Correspondingly, holes accumulate at the BTO/HZO interface, since the HZO layer remains insulating under illumination due to its optical transparency. This charge separation gives rise to a net downward E_PV_, as illustrated in Fig. 1b, which in turn drives the downward optical switching of polarization. It is important to emphasize that it has been reported that BTO ferroelectric order can vanish by the application of light [26, 27, 51, 52].

To test the proposed scenario for achieving optical switching of the polarization in structures based on ferroelectric HfO_2_, here we investigated a series of BTO capped HZO samples variable BTO layer thickness. The HZO/BTO bilayers were deposited on LSMO electrodes on STO substrates by PLD. We show that BTO grows in polycrystalline form on top of the epitaxial HZO. Endurance properties are reported to be robust and switching time fast with and without illumination. Optical switching of polarization occurs in samples where the BTO layer is thick enough to allow abundant generation of photogenerated charges to produce polarization switching.

## Experimental

### Sample Fabrication

Five HZO/BTO bilayers, with HZO thickness 8 nm and BTO layer thickness ranging from 1.5 to 100 nm, were grown by PLD on top of the LSMO electrodes deposited on STO(001) substrates. Two HZO/BTO bilayers, with HZO thickness 2 and 4 nm and BTO layer thickness of 10 nm, were also grown for comparison. An additional bare HZO film of 8 nm and a single BTO film of 100 nm were grown to be used as references. The (001)-oriented LSMO bottom layer, with a thickness of 25 nm, was deposited under dynamic oxygen pressure (PO₂) of 0.1 mbar and at a substrate temperature (T_s_) of 700 °C. Subsequently, the HZO layer was deposited under the same PO_2_ of 0.1 mbar but at T_s_ of 800 °C. BTO layers were finally deposited on top, with PO_2_ of 0.02 mbar and at T_s_ of 700 °C. Platinum top electrodes, 20 nm thick and 20 µm in diameter, were deposited onto the samples using dc magnetron sputtering through a stencil mask and these are shown in top planar view in Supporting Informations.

### X-Ray Characterization

The crystal structure was analyzed using X-ray diffraction (XRD) with Cu Kα radiation on a Bruker D8 Discover diffractometer equipped with a point detector. A Bruker D8-Advance diffractometer, equipped with a two-dimensional detector was also used.

### Scanning Transmission Electron Microscopy

Atomic-scale structural analysis of selected films was performed by scanning transmission electron microscopy (STEM) in high-angle annular dark field (HAADF) imaging mode. A Thermo Fisher Titan 60–300 microscope equipped with a high brightness Schottky field emission gun and a CETCOR probe-corrector (CEOS Gmbh) was operated at 300 kV to provide a probe size below 0.1 nm. Cross-sectional lamellae of the specimens, cut along (110) planes of the STO substrate, were prepared by focused ion beam milling in a Thermo Fisher Helios 650 Nanolab.

### Ferroelectric Characterization

Ferroelectric polarization and current versus voltage loops and endurance were measured at room temperature using an AixACCT TFAnalyser3000 platform in a top–bottom configuration [53], with the bottom electrode grounded and the top electrode biased. To compensate leakage in polarization loops, the dynamic leakage current compensation (DLCC) and positive-up-negative-down (PUND) methods were employed [54, 55]. Endurance tests were carried out using 1 MHz bipolar electric field pulses, with remanent polarization determined positive and negative polarization values from DLCC loops measured at 1 kHz at the same electric field. The pulse train used for switching dynamics experiments is described as follows. Firstly, a preswitching pulse is applied. This is long enough to ensure the polarization saturation at indicated in the manuscript electric field. Afterwards, a trapezoidal switching of rise/fall/plateau time (*τ*_w_) pulse is applied. After 1 s, a modified PUND pulse train is used for polarization state reading (ΔP) [56]. The leakage current was measured using 2 s integration time, with data averaged during both increasing and decreasing voltage sweeps. In the experiments performed under illumination, the device under test is continuously illuminated by light.

### Piezoelectric Force Microscopy Characterization

Piezoelectric force microscopy (PFM) measurements were taken with an MFP-3D microscope (Oxford Instruments Co.) using BudgetSensors silicon (n-type) cantilevers with Pt coating (Multi75E-G). To enhance sensitivity, the dual AC resonance tracking (DART) method was employed [64]. To remove charging effects contribution additional bias voltage was also employed during PFM characterization [65].

### Optical Excitation

Optical illumination during or after electrical tests or PFM tests was performed with a blue-violet laser source (λ = 405 nm, E_photon_ = 3.06 eV, power density ≈3.9 W cm^−2^). Note that at this wavelength the 20 nm thick Pt top electrode transparency is around 15% [57]. The incidence angle was fixed for all the measurements at ≈ 45°. Note that the energy of the used light (blue-violet; 3.06 eV) is below the bandgap energy of BTO (whose bandgap in bulk is about 3.3 eV [58]), and then band-to-band excitation cannot be produced. However, in BTO films, it is known that significant photon absorption can occur for sub-bandgap photon energies [59–61]. This is a result of the presence of oxygen vacancies or other point defects in BTO introducing allowed states within the bandgap [59, 62, 63].

### First-Principles Calculations

First-principles calculations based on DFT [66]. were carried out with the PBEsol exchange–correlation energy functional [67] as it is implemented in the VASP software [68]. The “projector-augmented wave” method [69] was employed to represent the ionic cores by considering the following electronic states as valence: Hf 5*d* 6*s* 5*p*; Zr 4*d* 5*s* 4*p*; O 2*s* 2*p*; Ba 5*s* 5*p* 6*s*; Ti 3*d* 4*s* 3*p*. An energy cutoff of 650 eV and a dense Monkhorst–Pack k-point density (equivalent to that of a 12 × 12 × 12 grid for the 12-atom bulk HfO_2_ unit cell) was used for integration within the Brillouin zone, leading to total energies converged to within 1 meV per formula unit. Atomic relaxations were concluded when the forces in all the atoms were below 0.005 eV Å^−1^. The simulated HZO slab contained a total of 48 atoms (16 Hf and 32 O ions), extending over a length of approximately 40 Å perpendicular to its surface (with a 20 Å thick vacuum region). Similarly, the simulated BTO slab contained a total of 160 atoms (32 Ba, 32 Ti and 96 O ions), extending over a length of approximately 50 Å perpendicular to its surface (with a 25 Å thick vacuum region). The materials band gaps were calculated using the range-separated hybrid functional HSE06 [48]. To estimate band alignments, we followed the computational approach described elsewhere [49, 50].

## Results

### Hf_0.5_Zr_0.5_O_2_/BaTiO_3_ Heterostructure Integration

**Fig. 2:**
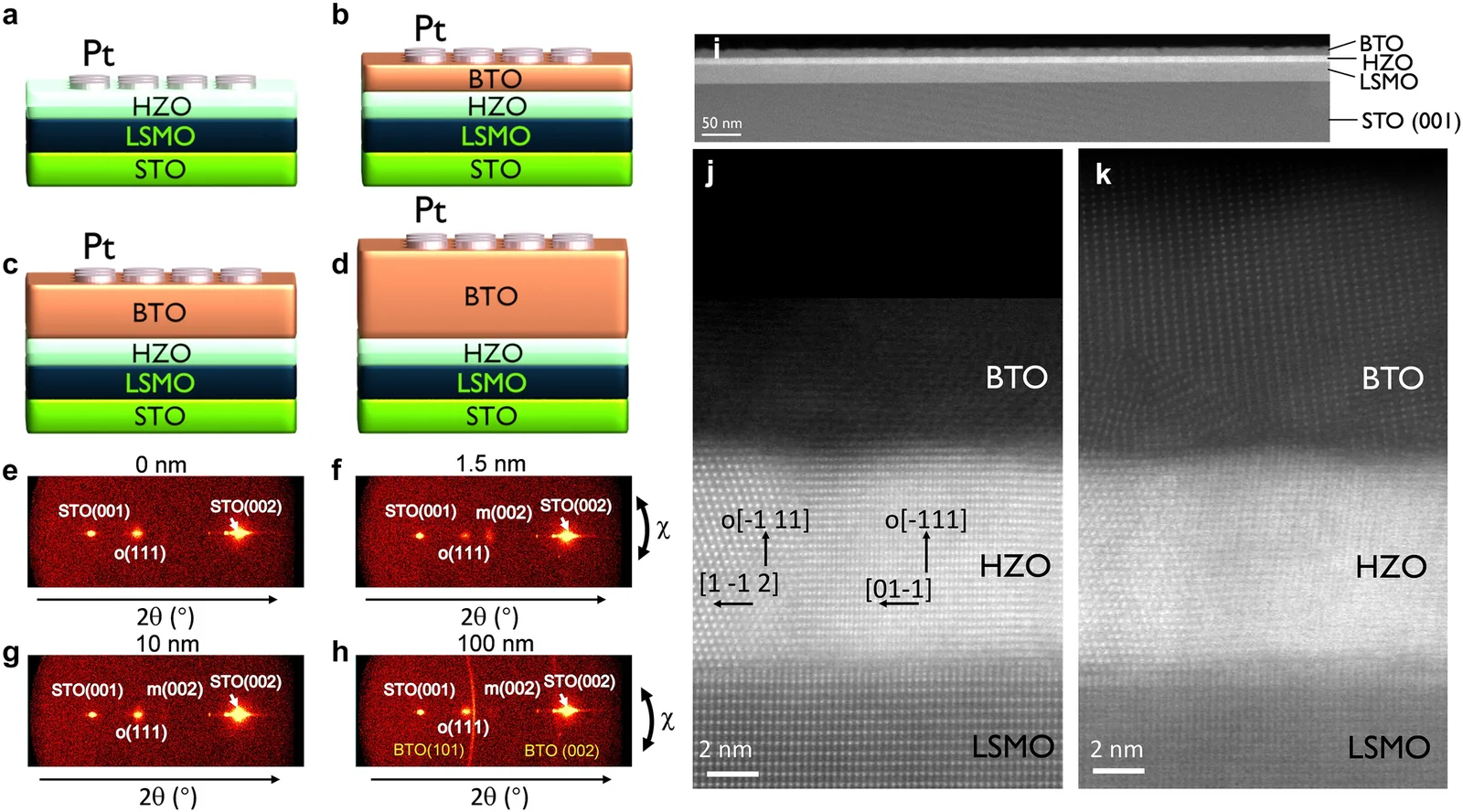
**a-d** Schematic representation of the representative HZO/BTO samples with BTO thickness of 0, 1.5, 10, and 100 nm, respectively. **e–h** XRD 2*θ*–χ maps of HZO/BTO samples with BTO thickness of 0, 1.5, 10, and 100 nm, respectively. **i** Low magnification cross sectional HAADF-STEM image corresponding to the sample with a 10-nm-thick BTO layer. **j, k** Atomic resolution HAADF images of representative regions for the same sample. In image **k** the focus was optimized to maximize the contrast of the lattice fringes in crystalline BTO grains, to the detriment of the LSMO-HZO interface

Representative HZO/BTO bilayers with 0, 1.5, 10, and 100 nm for the BTO are sketched in Fig. 2a-d, respectively. Platinum circular top electrodes were deposited onto the whole set of samples with 0, 1.5, 5, 10, 30, and 100 nm for the BTO layer, and these are shown in Fig. S1. HZO thicknesses are ≈ 8 nm for all films as extracted from the simulation of the Laue fringes in the *θ*-2*θ* XRD scans (Fig. S2). The BTO thicknesses were calculated from calibration of the growth rate. XRD 2*θ*-*χ* maps for the single-layer and the HZO/BTO representative samples of thickness 0, 1.5, 10, and 100 nm for the BTO layer are shown in Fig. 2e-h, respectively. The spots of highest intensity correspond to STO(001) and STO(002). The presence of the spot at 2*θ* ≈ 30° indicates the formation of the orthorhombic phase with (111) orientation. Monoclinic (m) (002) elongated spot (2*θ* ≈ 35°) is present in the sample capped with 1.5 nm BTO (Fig. 2f). The elongation of the spot along *χ* indicates a higher mosaicity of monoclinic crystals compared with the orthorhombic ones. The intensity of this diffraction spot is lower in the other samples. In the sample capped with 100 nm BTO (Fig. 2h), the arc corresponding to BTO(101) (2*θ* ≈ 31°) crystallites indicates its polycrystalline growth. Integrated around *χ* ≈ 0°, 2*θ* scans are shown in Fig. S3. Figure 2i shows a low-magnification HAADF-STEM image with a field of view of about 800 nm of the film with a 10 nm BTO capped sample. The BTO surface exhibits a rougher morphology, consistent with its polycrystalline nature. Figure 2j presents an atomic resolution STEM analysis of the local microstructure of the HZO. The semi-coherent interface between the HZO film and the LSMO bottom electrode, resulting from the domain-matching epitaxy growth mechanism [40], is clearly visible. The interface appears relatively sharp, which is attributed to its chemical reconstruction resulting in its La-rich composition [70]. The HZO layer is predominantly composed of orthorhombic grains, oriented along the [111]-type out-of-plane direction in agreement with XRD characterization. In this case, the BTO appears out of focus due to slight differences in ion milling rate and amorphization layer during lamella preparation. On the other hand, in Fig. 2k BTO grains appear in focus, and are coincidentally close to the tetragonal [110] zone axis, which allows the crystallinity of BTO to be more clearly inferred. Semi-coherent growth of BTO on HZO is evident from the lattice alignment at the interface. The polycrystalline nature of BTO is better inferred in the wider image shown in Fig. S4.

**Fig. 3:**
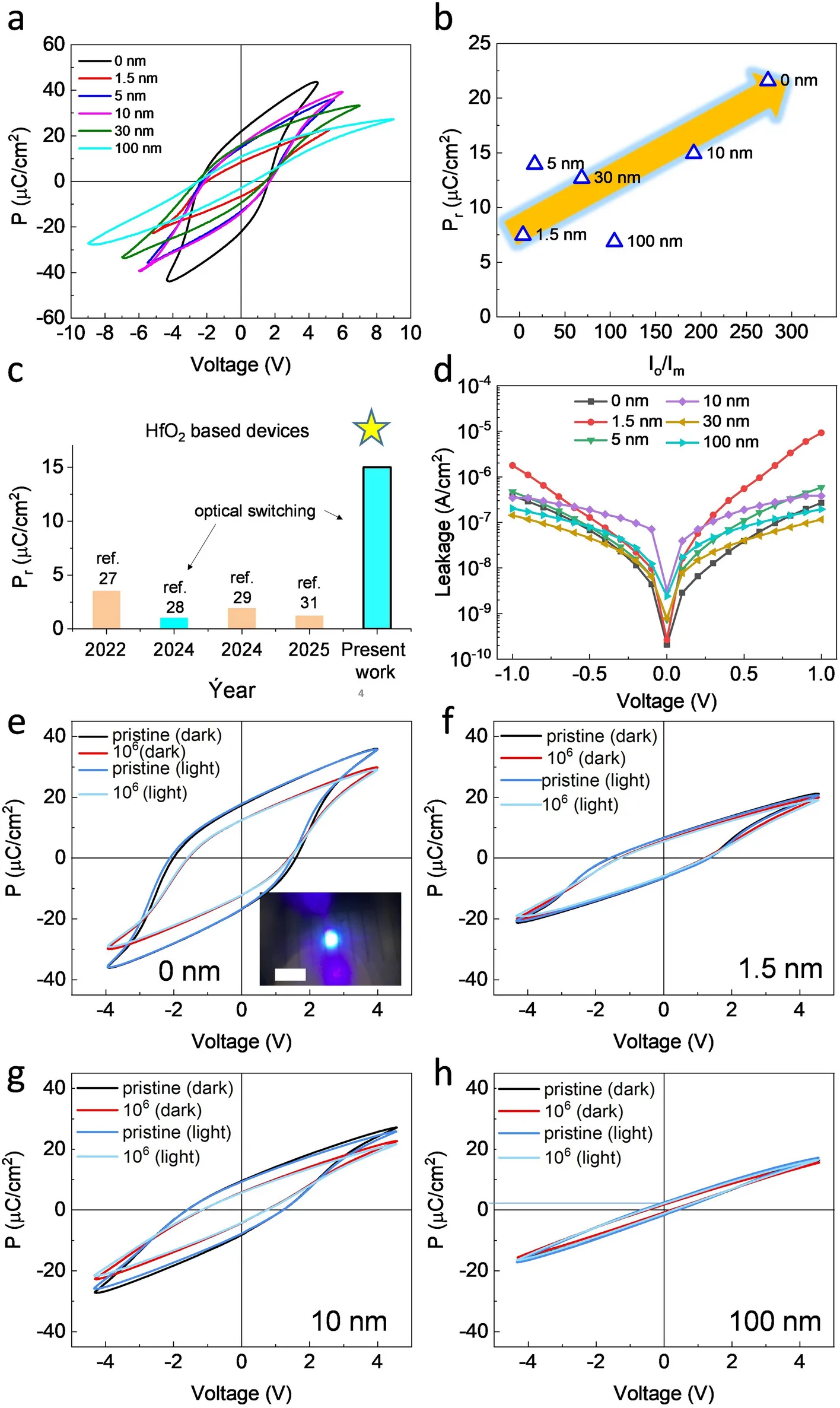
**a** P–V loops of samples with BTO thickness of 0, 1.5, 5, 10, 30, and 100 nm. **b** Dependence of P_r_ on the ratio of peak intensities of o(111) and m(002) reflections, extracted from Fig. S2. **c** Summary of P_r_ values extracted from the literature and the present work in systems based on HfO_2_ or ZrO_2_ showing optoelectric response to visible light. **d** Leakage current of samples with BTO thickness of 0, 1.5, 5, 10, 30, and 100 nm. **e–h** P–V loops of HZO/BTO representative samples with BTO thickness of 0, 1.5, 10, and 100 nm, respectively, with and without illumination and in the pristine state and after 10^6^ cycles

Polarization–Voltage (P–V) loops measured at saturation voltage for the whole set of samples show P_r_ values between 7 and 15 μC cm^−2^ and a correlation between P_r_ and the intensity of the o(111) diffraction peak normalized to the m(002), as shown in Fig. 3a, b, respectively. In Fig. S5, we show ferroelectric characterization of a 100 nm BTO film on LSMO/STO, nominally grown under the same conditions as BTO capping. It can be observed that as anticipated the coercive electric field is much smaller than for HZO layer (of 225 kV cm^−1^), along with sizeable (16 μC cm^−2^) and robust against cycling ferroelectric polarization and leakage current slightly larger than that shown for most of the HZO capped samples (0.4 μA cm^−2^ at 1 V). Intensities of the diffraction peaks are extracted from Fig. S2. Therefore, it can be concluded that P_r_ is dominated by the different stabilization of o(111) phase depending on BTO thickness. The presence of depolarization electric field generated by BTO [71] is not evident. Among the films capped with BTO, the largest P_r_ value (15 μC cm^−2^) is observed for the 10 nm BTO capped sample. Compared with other HfO_2_- or ZrO_2_-based systems that exhibit an optoelectric response [31–35], this value is clearly higher, as summarized in Fig. 3c and Table 1. Leakage curves shown in Fig. 3d display that leakage current does not significantly depend on thickness. It can be noted that the 1.5 and 10 nm BTO-capped samples exhibit slightly higher leakage, probably due to a larger number of defects arising from sample-to-sample variability. In fact, as mentioned, for BTO-capped films above 1.5 nm the leakage current at 1 V is smaller than 10^–6^ A cm^−2^, lower than those previously reported for uncapped HZO films of similar thickness [72] and close to the very low values obtained in epitaxial La-doped HZO films [73].

### Stable and Robust Ferroelectric Response

P–V measurements were taken at 4 V to better test the response upon cycling. At this voltage, sizeable switchable polarization is observed, even though full saturation polarization is not reached, which helps prevent breakdown. P–V loops of the single HZO layer and the samples with BTO capping of thicknesses 1.5, 10, and 100 nm are shown in Fig. 3e-h, respectively. P–V loops show that the P_r_ is 17.5 μC cm^−2^ for the non-capped sample, and it decreases to around 7.5 μC cm^−2^ for the 1.5 and 10 nm BTO-capped samples and to 2 μC cm^−2^ for the 100 nm-BTO capped sample. Clear ferroelectric switching peaks can be seen in I-V curves of the 0, 1.5, and 10 nm BTO capped samples, but less evident for the 100 nm one, as shown in Fig. S6. The reduction in polarization in BTO capped samples can be ascribed to the partial screening of the ferroelectric polarization of HZO, resulting from electron doping caused by the straddling band alignment at the HZO/BTO interface (Fig. 1d). Additionally, as shown in Fig. 3b, the orthorhombic phase fraction might also importantly influence P_r_. It should also be noted that the bare HZO films exhibit a polarization lower than theoretical predictions, which estimate values in the range of 52–55 ﻿μC cm^−2^ [74, 75]. Experimentally, this corresponds to approximately 32 μC cm^−2^ for a (111)-oriented, phase-pure orthorhombic HfO₂ film, as reported elsewhere [76–79] indicating that orthorhombic phase fraction is around 50%-60% in the characterized films. In Fig. 3e-h, P–V curves after 10^6^ cycles at 4 V are also shown. It can be observed that a similar response is observed among samples. Polarization variation upon cycling is small, disregarding the presence of any wake-up effect or significant fatigue. The loops were also recorded under illumination (see I-V curves in Fig. S6). The inset of Fig. 3a shows a picture of the used light spot. In brief, it can be observed that polarization, even after cycling, does not suffer significant variation under illumination, in agreement with the mentioned wide HfO_2_’s bandgap (≈ 6.0 eV) [29, 30].

**Fig. 4:**
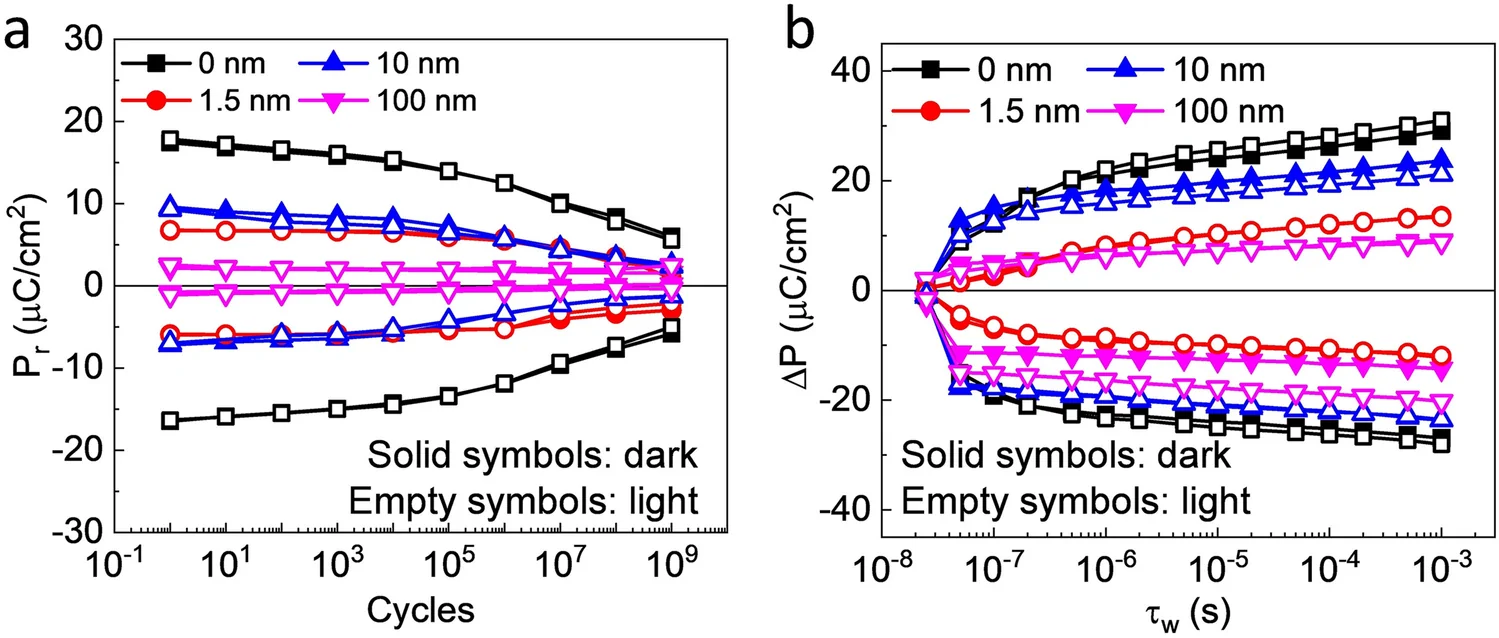
**a** Endurance measured at 1 MHz and 4.5 V cycling voltage for 1.5, 10, and 100 nm samples and at 1 MHz and 4 V for the bare HZO film in the dark (solid symbols) and with illumination (open symbols). **b** Switching spectroscopy plots at 4.5, 5, 6, and 9 V writing and reading amplitudes for 0, 1.5, 10, and 100 nm samples, respectively, in the dark (solid symbols) and with illumination (open symbols)

In Fig. 4a, the full endurance measurements are shown, and the corresponding raw data for all samples are shown in Fig. S7. Figure 4a, shows that the polarization is sizeable and stable upon large number of cycles (10^8^), except for 100 nm BTO that shows small P_r_ at the used cycling voltage and ferroelectric switching peaks can only be observed up to 10^7^ cycles (Fig. S7). Figure 4a also shows the endurance data collected under illumination. The values are very similar to those obtained in the dark, further confirming the robustness of the ferroelectric character of the characterized samples. The absence of light effect on the ferroelectric endurance reveals, in addition to the robust ferroelectric response, the absence of heating effects, as far as endurance in hafnia is highly sensitive to temperature [80]. Switching spectroscopy data are shown in Fig. 4b. It can be observed that the *τ*_w_ onset is near 50 ns, limited by the set-up time-constant. For increasing *τ*_w_, P_r_ increases without full saturation, due to the presence of non-ferroelectric contributions at large *τ*_w_ [56]. *τ*_w_-dependence under illumination also shows similar results, with variations inside the junction to junction variance. Raw data for switching spectroscopy are shown in Fig. S8.

### Optical Switching of Polarization

**Fig. 5:**
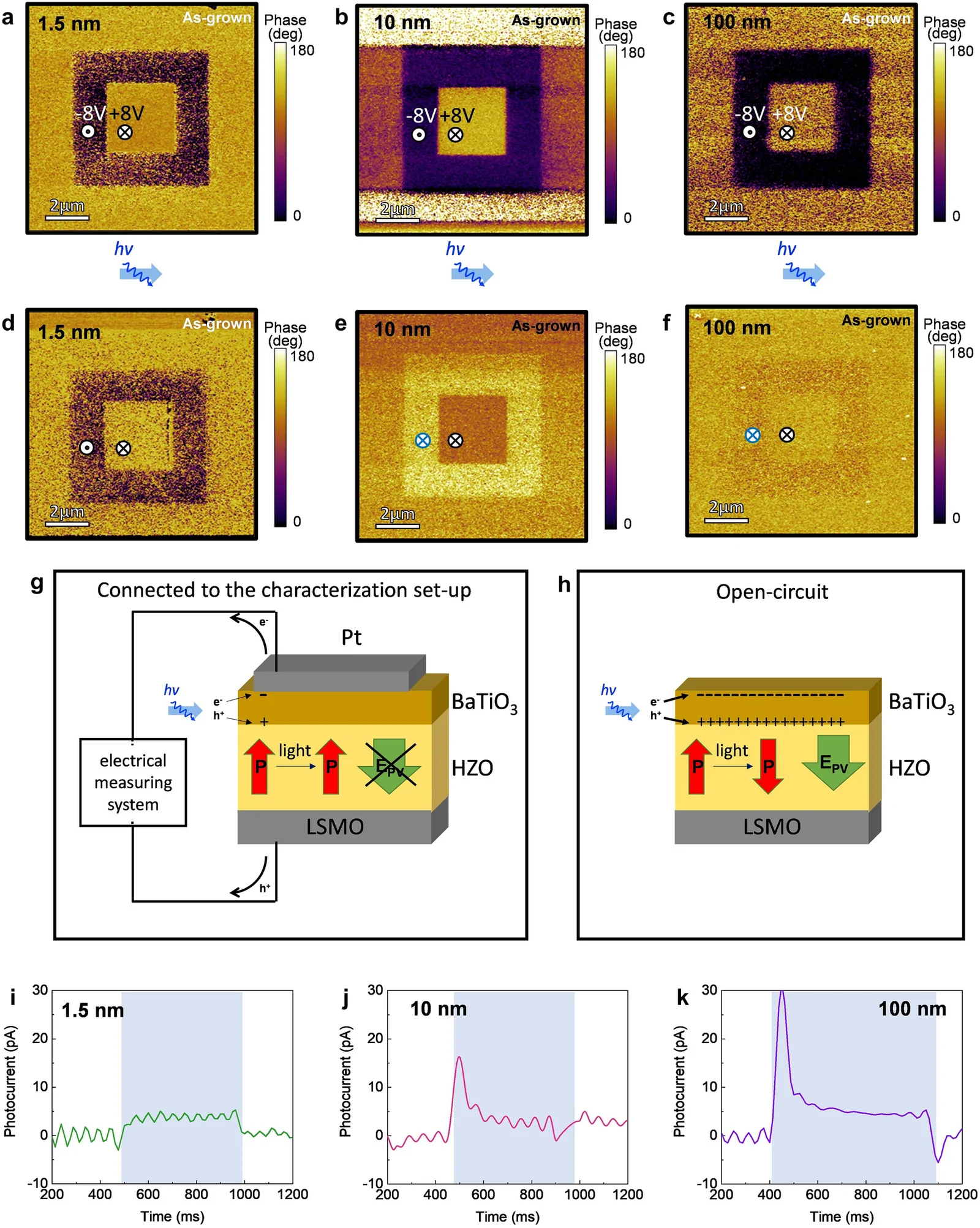
PFM phase images after** a-c** electrical poling and waiting for 60 s in the dark and **d-f** after illuminating during 60 s for 1.5, 10, and 100 nm samples, respectively. Arrows indicate the polarization state and voltage the electrical writing voltage. In (**d-f**) blue arrows indicate optical polarization switching. **g, h** Sketch of the different charge flow behavior of the system while performing macroscopic ferroelectric characterization using Pt top electrodes connected to the measuring set-up and while performing PFM characterization without Pt top electrodes in open-circuit conditions, respectively. **i-k** I_SC_ dependence on time for 1.5, 10, and 100 nm samples, respectively. Bluish background indicates the timeframe when the sample was illuminated

All in all, the results presented up to here indicate that the presence of BTO layer causes a decrease of P_r_, while a good endurance and fast switching are observed, both robust under illumination. Figure 5a-c shows PFM phase maps for the 1.5, 10, and 100 nm-thick samples collected on the BTO surface (without Pt), respectively, obtained after applying -8 V in the darker region and + 8 V in the brighter inner region. There is 180° phase contrast in all cases, indicating that polarization is pointing upwards in the dark region and downwards in the bright region, as indicated. Outer region corresponds to the as-grown state, which shows similar contrast to that obtained after + 8 V, and thus the as-grown samples present a downwards state. Figure 5d-f corresponds to PFM phase maps collected in the same region after illumination. It can be observed that for the 1.5 nm-BTO sample the contrast almost remains. Only some bright spots can be observed in the initially upwards region. This is not the case for 10 and 100 nm capped samples, where absence of 180° phase contrast is observed, indicating that in the dark regions of Fig. 5b, c, the polarization has switched from upwards to downwards. This indicates that the optical switching of polarization occurs more efficiently for thicker BTO layer. This agrees with the expected larger photogenerated charges due to larger light absorption, if the BTO-capping layer is thicker, which results in large E_PV_ (Fig. 1) and thus a more efficient optical switching of polarization. Indeed, Fig. S9 shows that polarization is not switched even after 10 min of illumination in same conditions for a non-capped sample. The residual contrast after illumination is because extrinsic charging effects can remain. Corresponding PFM amplitude images of Fig. 5 are shown in Fig. S10. PFM phase images collected after various illumination times are shown in Fig. S11 and allow to conclude that for 30 s illumination most of the ferroelectric domains that were pointing upwards have switched downwards. Instead for 1 s the polarization switching is partial, indicating that using the given conditions switching time is around or near above 1 s, which is comparable to switching time reported in other systems based on hafnia [32] as summarized in Table 1. Figure S12, where equivalent experiments are shown for 2 and 4 nm HZO layers with 10 nm BTO on top, shows that HZO layer thickness do not significantly impact on the observed optical polarization switching as expected by the fact that ferroelectricity in epitaxial HZO is preserved down to the ultrathin limit [81, 82]. Note that for the ferroelectric characterization shown in Figs. 3 and 4, the Pt top electrode is present and the device is connected to the electrical measurement system. As a result, photogenerated carriers in the BTO layer do not accumulate at the interface but instead flow through the circuit (Fig. 5g). Consequently, E_PV_ is not generated. In contrast, during PFM characterization the sample is in open-circuit conditions, so charges can only recombine or accumulate at the interfaces. This leads to the generation of E_PV_, which drives the optically induced polarization switching (Fig. 5h), as anticipated in the description of Fig. 1b. Indeed, Fig. 5i-k shows the short-circuit photocurrent (I_SC_) response measured using Pt top electrodes for the 1.5, 10, and 100 nm HZO/BTO samples, respectively. It can be observed that although the response is small, it is sizeable. As expected, I_SC_ increases with BTO thickness, consistent with the higher absorption of thicker films as described by Beer-Lambert’s law and with the absence of optical switching for the 1.5 nm BTO sample. It can be also observed that the current is positive indicating that the electric field is directed downwards (as depicted in Figs. 1b and 5h) thus dictating the final state after illumination as downwards. The current peaks, at the moment of illumination start–end, if present, correspond to charging and discharging processes. No significant modulation of the I_SC_ with the preset polarization state is observed. A similar absence of I_SC_ dependence on polarization has been previously reported in systems based on different ferroelectric materials [53, 83–85], highlighting the predominant role of internal (built-in) electric fields over those associated with ferroelectric polarization. Indeed, the final polarization state is consistently oriented downwards irrespectively of the initial polarization sign, and the modification of the final state would only be possible through modification of the internal electric fields, as reported elsewhere [28]. In summary, we have demonstrated that the BTO capping layer plays a key role due to its high polarizability and its efficiency as a light absorber. Doped BiFeO₃ constitutes an interesting alternative, as it combines a comparatively reduced bandgap (≈2.7 eV) with a large remanent polarization in the range of 60–100 ﻿μC cm^−2^ [86–89]. In contrast, Pb_x_Zr_1-x_TiO_3_, another archetypal ferroelectric, is less suitable due to its larger bandgap (> 3.5 eV) [90, 91]. It should be noted, however, that both material families contain Bi and Pb, which are considered toxic and therefore limit their prospects for technological applications, despite their relevance for fundamental studies. Hexagonal manganites, such as YMnO_3_ and LuMnO_3_ [83, 92, 93], represent another interesting alternative, as they exhibit very narrow bandgaps (≈1.5 eV). Nevertheless, their relatively low polarization (≈5 μC cm^−2^), substantially smaller than that of HZO, may be detrimental to the overall ferroelectric response. Exploring the broad materials family of two-dimensional ferroelectrics is also a promising research direction, as these systems can display a wide range of bandgaps, although they typically also exhibit low remanent polarization values [94, 95]. Non ferroelectric materials such as TiO_2_, which exhibits a bandgap at the edge of the visible spectrum (E_g_ = 3.05 eV [96]) and a high permittivity (ranging from 30 to 120 depending on the stabilized phase [97, 98]), can also be considered promising candidates for similar applications.

## Conclusions

We have shown that the HZO/BTO system presents robust ferroelectric properties with fast switching response, being the BTO thickness of 10 nm optimal. The crystalline quality of both HZO and BTO layers account for the observed good ferroelectric properties, i.e., P_r_ = 15 μC cm^−2^, leakage current under 10^–6^ A cm^−2^ at 1 V, endurance up to 10^8^ cycles and measured response time as fast as 50 ns (limited by the set-up). This performance exceeds that of previously reported HfO_2_- and ZrO_2_-based systems exhibiting optoelectric responses (Table 1). The sizeable photoresponse allows the optical switching of the polarization, as confirmed by PFM. In the studied system, BTO is responsible for the light absorption and photocarrier generation. Our findings establish a clear pathway for the development of high-performance photoferroelectric memory devices based on ferroelectric HfO_2_.

## Supplementary Information

Below is the link to the electronic supplementary material.

Supplementary file1 (DOCX 36192 kb)
