# Supplementary material for: Optical Switching of Robust Ferroelectric Polarization on Epitaxial Hf0.5Zr0.5O2 Integrated with BaTiO3
Source: Nanomicro Lett. 2026 Feb 6;18:239. doi: 10.1007/s40820-026-02090-2 (PMC12876513; doi:10.1007/s40820-026-02090-2)
Supplement: Supplementary file 1 — Supplementary file1 (DOCX 36192 kb) [file 40820_2026_2090_MOESM1_ESM.docx]

Supporting Information for

Optical Switching of Robust Ferroelectric Polarization on Epitaxial Hf_0.5_Zr_0.5_O_2_ Integrated with BaTiO_3_

Wenjing Dong^1^, Huan Tan^2^, Jingye Zou^1^, Alberto Quintana^1^, Tingfeng Song^1^, César Magén^3,4^, Claudio Cazorla^5,6,7^, Florencio Sánchez^1,*^ , Ignasi Fina^1,*^

^1^Institut de Ciència de Materials de Barcelona (ICMAB-CSIC), Campus UAB, Bellaterra 08193, Barcelona, Spain

^2^Departament de Física, Universitat Autònoma de Barcelona, 08193 Cerdanyola del Vallès, Spain

^3^Instituto de Nanociencia y Materiales de Aragón (INMA), CSIC-Universidad de Zaragoza, 50009 Zaragoza, Spain

^4^Departamento de Física de la Materia Condensada, Universidad de Zaragoza, 50018 Zaragoza, Spain

^5^Group of Characterization of Materials, Departament de Física, Universitat Politècnica de Catalunya, Campus Diagonal Besòs, Av. Eduard Maristany 10–14, 08019 Barcelona, Spain

^6^Research Center in Multiscale Science and Engineering, Universitat Politècnica de Catalunya, Campus Diagonal-Besòs, Av. Eduard Maristany 10–14, 08019 Barcelona, Spain

^7^Institució Catalana de Recerca i Estudis Avançats (ICREA), Passeig Lluís Companys 23, 08010 Barcelona, Spain

*Corresponding authors. E-mail: [ifina@icmab.es](mailto:ifina@icmab.es) (Ignasi Fina); [fsanchez@icmab.es](mailto:fsanchez@icmab.es) (Florencio Sánchez)

**Supplementary Figures and Tables**


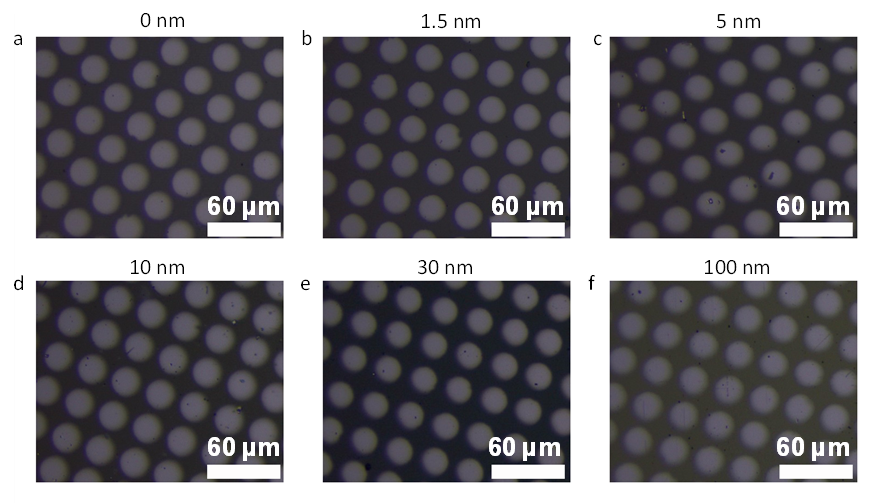


**Fig.** **S1** (**a-e**) Optical image (top view) of the deposited Pt electrodes of the samples with BTO thickness of 0, 1.5, 5, 10, 30 and 100 nm, respectively

**Fig.** **S2** (**a-e**) XRD θ –2 θ scans (measured with a point detector ) of the samples with BTO thickness of 0, 1.5, 5, 10, 30 and 100 nm, respectively

**Fig. S3** (**a-l**) XRD 2 θ –χ images and integrated, in the χ = ±10° range around χ = 0°, θ–2θ scans of samples with BTO thickness of 0, 1.5, 5, 10, 30 and 100 nm as indicated. The † and * symbols mark the positions of the STO(002) reflections due to spurious Cu-K_β_ and W-L lines, respectively

**Fig.** **S4** Atomic resolution HAADF images of a representative region where the good crystalline quality of the BTO layer can be observed

**Fig.** **S5** (**a**) P-V loops for 100 nm BTO sample without HZO at increasing voltage. (**b**) Leakage current for 100 nm BTO sample. (**c**) Endurance plots at 1 MHz and 10 V cycling voltage for 100 nm BTO sample

**Fig.** **S6** (**a-d**) Corresponding to Fig. 3(a-d), I-V loops of HZO/BTO samples with BTO thickness of 0, 1.5, 10 and 100 nm, respectively with and without illumination and in the pristine state and after 10^6^ cycles

**Fig.** **S7** (**a-e**) Endurance plots at 1 MHz and indicated voltage of samples with BTO thickness of 0, 1.5, 5, 10, 30 and 100 nm. (**f-k**) P-V and **(l-q**) I-V loops corresponding to data of **a-e**

**Fig. S8** (**a-d**) Polarization in dark and (**e-h**) under illumination loops for reading in the switching spectroscopy experiments. Asymmetric loops account for the presence of imprint electric fields

**Fig.** **S9** (**a**) PFM amplitude and (**b**) phase images collected just after poling, respectively, for a HZO single film without BTO. (**c, d**) Idem collected after waiting 10 min (600 s) under illumination

**Fig.** **S10** PFM amplitude images after (**a-c**) electrical poling and waiting for 60 s in dark and (**d-f**) after illuminating the sample for 60 s for 1.5, 10 and 100 nm samples, respectively

**Fig.** **S11** (**a**) PFM phase images collected after indicated illumination time. Arrows indicate the preset polarization state direction. (**b**) Phase profiles after indicated illumination time obtained from averaging images of panel (a) along vertical axis. (**c**) DPhase as a function of illumination time evaluated as the phase contrast of phase profiles in panel (b)

**Fig.** **S12** PFM amplitude and phase images collected just after poling and after 60 s under illumination for (**a**) 2 nm and (**b**) 4 nm HZO samples capped with 10 nm BTO
